# A continent-wide detailed geological map dataset of Antarctica

**DOI:** 10.1038/s41597-023-02152-9

**Published:** 2023-05-18

**Authors:** Simon C. Cox, Belinda Smith Lyttle, Samuel Elkind, Christine Smith Siddoway, Paul Morin, Giovanni Capponi, Tamer Abu-Alam, Matilda Ballinger, Lauren Bamber, Brett Kitchener, Luigi Lelli, Jasmine Mawson, Alexie Millikin, Nicola Dal Seno, Louis Whitburn, Tristan White, Alex Burton-Johnson, Laura Crispini, David Elliot, Synnøve Elvevold, John Goodge, Jacqueline Halpin, Joachim Jacobs, Adam P. Martin, Eugene Mikhalsky, Fraser Morgan, Phil Scadden, John Smellie, Gary Wilson

**Affiliations:** 1 GNS Science, Private Bag 1930, Dunedin, 9054 New Zealand; 2 Department of Geology, Colorado College, Colorado Springs, CO 80903 USA; 3 Polar Geospatial Center, University of Minnesota, St Paul, MN 55108 USA; 4 DISTAV, Università degli Studi di Genova, Corso Europa 26-16132, Genova, Italy; 5 Norwegian Polar Institute, P O Box 6606 Stakkevollan, N-9296 Tromsø, Norway; 6 Institute for Marine and Antarctic Studies, University of Tasmania, Private Bag 129, Hobart, TAS 7001 Australia; 7 University of Edinburgh, Old College, South Bridge, Edinburgh, EH8 9YL UK; 8 University of Otago, P O Box 56, Dunedin, 9054 New Zealand; 9 British Antarctic Survey, High Cross, Madingley Road, Cambridge, CB3 0ET UK; 10 Ohio State University, 125 South Oval Mall, Columbus, OH 43210 USA; 11 Department of Earth and Environmental Sciences, University of Minnesota Duluth, Duluth, MN 55812 USA; 12 University of Bergen, PO Box 7803, NO-5020 Bergen, Norway; 13 Gramberg Institute for Geology and Mineral Resources of the World Ocean (VNIIOkeangeologia), Angliiskii pr. 1, St Petersburg, 190121 Russia; 14 Manaaki Whenua Landcare Research, Private Bag 92170, Auckland, 1142 New Zealand; 15 University of Leicester, University Road, Leicester, LE17RH UK; 16 GNS Science, P O Box 30368, Lower Hutt, 5040 New Zealand; 17 Present Address: Department of Arctic and Marine Biology, UiT The Arctic University of Norway, P O Box 6050 Langnes, N-9037 Tromsø, Norway; 18 Present Address: Great Boulder Resources, 51 Colin Street, West Perth, WA 6005 Australia

**Keywords:** Geology, Geomorphology

## Abstract

A dataset to describe exposed bedrock and surficial geology of Antarctica has been constructed by the GeoMAP Action Group of the Scientific Committee on Antarctic Research (SCAR) and GNS Science. Our group captured existing geological map data into a geographic information system (GIS), refined its spatial reliability, harmonised classification, and improved representation of glacial sequences and geomorphology, thereby creating a comprehensive and coherent representation of Antarctic geology. A total of 99,080 polygons were unified for depicting geology at 1:250,000 scale, but locally there are some areas with higher spatial resolution. Geological unit definition is based on a mixed chronostratigraphic- and lithostratigraphic-based classification. Description of rock and moraine polygons employs the international Geoscience Markup Language (GeoSciML) data protocols to provide attribute-rich and queryable information, including bibliographic links to 589 source maps and scientific literature. GeoMAP is the first detailed geological map dataset covering all of Antarctica. It depicts ‘known geology’ of rock exposures rather than ‘interpreted’ sub-ice features and is suitable for continent-wide perspectives and cross-discipline interrogation.

## Background & Summary

Antarctica contains minimal geologic exposure compared to the overall area of ice, but there are over 52,000 km^2^ of rock and unconsolidated cover deposits that contain a rich geological, geomorphological and glaciological history of the continent. Numerous, hard-copy, regional-scale geological maps were developed last century^1–3^. Many have been scanned, some have been georeferenced, but few are more than raster digital information with an adjacent legend. For the most part they are geologically reliable for defining bedrock geology (‘deep time’) and construction of the continent. But the maps have poor spatial reliability in the context of modern science located by global positioning system (GPS) and other satellite sensors, and existing maps rarely contain much representation of glacial geology and cover sequences that hold information on the waxing and waning of Antarctica’s ice sheets.

A strong imperative for a comprehensive digital dataset of Antarctica’s geosphere comes from the need to understand its influence on global climate and potential contributions to sea level rise, as well as the effects of climate change on the frozen continent itself^4,5^. This drives interest in a geospatial resource that can pinpoint the locations of glacial deposits, indicate their mode of formation, age, and likely source. Although there are some relatively small areas represented by detailed local maps^6–8^ there are no modern attribute-rich geographic information system (GIS) datasets to provide holistic information commensurate with the scale of the ice sheets/ice shelves. Spatial variations in geological characteristics, such as density, colour, heat-production or sub-ice structure, are also now deemed to be important as they influence ice sheet dynamics and the response of the continent to ice-removal^9–12^. Geological data also provide contextual information for biological and ecological analysis, studies of albedo and meltwater production, soil conservation and managing human impact^13–16^. Meanwhile, large quantities of satellite data are being rapidly acquired at sub-metre scale over most of the Antarctic continent, offering the opportunity to locate outcrops and derive high-resolution compositional information where data can be referenced to ground-based observations^17–19^.

Following publication of the 1:250,000 Geological Map of New Zealand^20,21^ and release of a map sheet and GIS for southern Victoria Land^22^, GNS Science launched an ambitious project to build a similar high-quality digital geological dataset covering the entire Antarctic continent. International participation and support were sought through formation of a Geological Mapping Update of Antarctica (GeoMAP) Action Group at the 2014 Scientific Committee on Antarctic Research (SCAR) Open Science Conference in Auckland (https://www.scar.org/science/former-groups/geomap/). The aim was to capture existing geological map data, update its spatial reliability, improve representation of glacial sequences and geomorphology, then enable data delivery via web-feature services. Our broader intent is to provide a dataset describing the exposed geosphere that can be used for cross-discipline science, as well as continent-wide geological perspectives^2,3,23^.

The GeoMAP Action Group attracted principal collaborators from United States of America, Norway, Italy, United Kingdom, Australia, Russia and New Zealand, but included contributions from at least 14 nations. Many others provided advice, data and support (see Acknowledgements). Much manual work was completed in the GNS Science office in Dunedin by 11 student volunteers, who visited New Zealand on internships or worked remotely by videoconferencing in return for GIS-training and professional development. Students presented the results of their local mapping in conference abstracts, talks and posters as the dataset progressively evolved and improved. An initial beta-test version of GeoMAP (v.2019-07) was released at the 2019 XIII International Symposium of Antarctic Earth Sciences meeting in Korea. Following a period for review and further improvements by GNS Science, the first formal version has been released and is documented here.

**Fig. 1:**
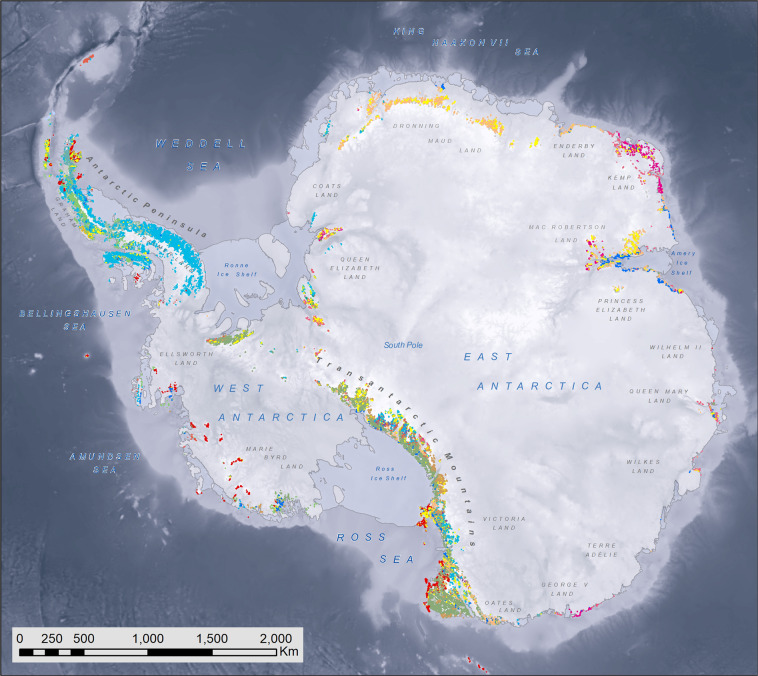
A geological map of Antarctica generated using the GeoMAP dataset, here draped over subglacial topography from MEaSUREs BedMachine Antarctica^11,55^. A rich attribute table enables data to be displayed or queried in a wide variety of ways. Here the map renders 99,080 polygons with colours reflecting rock or deposit age using MAPSYMBOL according to the legend in Fig. 2, many of which are too small to be seen at a continent scale. Other data captured in GeoMAP, but not displayed on this map, includes a source bibliography and fault data.

The new GeoMAP v.2022-08 dataset of Antarctic geology (Fig. 1) is now freely available from the World Data Center PANGAEA (10.1594/PANGAEA.951482)^24^. It describes and presents the ‘known geology’ of rock and bare sediment exposures in a unified framework. As well as providing a fundamental dataset for local- to continental-scale interrogations of bedrock and glacial geology together with glaciology and other physical sciences, the nature and composition of rock substrate as defined by GeoMAP also provides a contextual base for biological and ecological research.

## Methods

### Goals and context

The GeoMAP Action Group set itself a challenge to collaboratively build a modern geological dataset to classify and describe the bedrock and surficial deposits representing Antarctica’s geology. The concept was to capture existing geological observations and map data, update its spatial reliability, develop data in a GIS format and enable data delivery via web-feature services. Providing links to all original publications through a spatial bibliography was deemed paramount in creating a fair and functional product. The goal was to provide a dataset describing the exposed geosphere which would enable cross-discipline science or, for continent-wide perspectives, that captured existing geological knowledge in a manner that can be easily improved by subsequent generations. A secondary goal was to improve representation of glacial sequences and geomorphology because of their potential to contain records of ice fluctuations relevant to climate change.

Rapid development of a continent-wide dataset was achieved by adapting a tried and tested GIS methodology from mapping New Zealand (QMAP 1993–2014)^20,25^ but adopting a distinct ‘downscaling’ work-stream from continental- to regional- to local-scale. This differs from the more-traditional and common approach of dividing a region of interest into a series of mapsheet areas that are completed sequentially at the same scale. Construction of GeoMAP started from a continent-scale, low density, attribute-poor dataset that was augmented and improved through multiple iterations. The work-flow followed a seven stage process adapted from the digital geological map of New Zealand: (1) adjusting rock and moraine polygons; (2) scanning and registering old maps to build a spatial bibliography; (3) coding polygons with attributes according to legacy map classification and source information; (4) assigning continent-wide classification and building a consistent legend (the hardest part); (5) reviewing depiction of glacial geology and cover sequences; (6) translating into the Geoscience Markup Language (GeoSciML) data standard^26^ published by the International Union of Geological Sciences (IUGS) Commission for Geoscience Information (https://cgi-iugs.org/project/geosciml/); and (7) constructing a seamless continent-wide dataset, with associated reviewing, checking and quality control.

A key feature was the deliberate strategy to capture ‘known geology’ of rock and bare sediment exposures rather than ‘interpreted’ sub-ice features. In practice this meant classifying and describing around 52,000 km^2^ of the continent, now distinguished by 99,080 distinct polygons. These have been unified for use at approximately 1:250,000 scale, although locally there are some areas with higher spatial precision. Feature classification and description of rock and moraine polygons utilised a mixed chronostratigraphic- and lithostratigraphic-based classification, which employs the international GeoSciML 4.1 XML-based data protocols^26^ to provide attribute-rich and queryable data, including bibliographic links to the source maps and literature where original field observations were published. Although there are varying degrees of interpretation within any map at a local-scale, from a continental-perspective GeoMAP is more of an ‘observational dataset’ or discontinuous ‘fact map’, rather than a more-continuous ‘interpretational dataset’.

### Input data

Areas depicting rock were initially derived from the SCAR Antarctic Digital Database (ADD) v5.0 shapefile Rock_outcrop_high_res_polygon.shp^27^. Although there are known problems locally with the georeferencing, overestimation and generalisation^28^, this dataset of 68,381 polygons provided a pragmatic balance between a continent-wide dataset with enough precision and accuracy to enable regional-scale work, yet it is not so large as to be unworkable on a standard desk-top computer. Rock outcrop and lake polygons were examined against aerial photographs, the Landsat Image Mosaic of Antarctica (LIMA) imagery^29^ and against outputs from high-resolution satellite products and automated algorithms^18,28,30^. Those polygons that were particularly poorly georeferenced (with >250 m error), or misshaped, were manually adjusted to concur with LIMA, which has 15 m panchromatic pixels geolocated to ±54 m one-sigma accuracy^29^. Mapping of unconsolidated Neogene cover deposits involved checking a combination of previous work and the ADD shapefile Antarctic_moraines_high_res_polygon.shp^27^, then making new interpretations from aerial photographs, satellite imagery, light detection and ranging (LiDAR) elevation data and the Reference Elevation Model of Antarctica (REMA)^29–31^.

Geological observations were compiled from 589 sources, which include a wide variety of published maps^1–3^ and papers^6,8,17^, unpublished maps, university theses and other compiled and already available digital data^22,32^. Relocated and reshaped ‘rock’ and ‘moraine’ polygons were further cut and/or reshaped to represent variations and features of geology depicted in the source maps. Some areas have detailed mapping at scales of less than 1:50,000 whereas others are mapped more regionally at 1:250,000. Of the 589 maps scanned and georeferenced, 49% are local-scale (<1:250,000), 44% regional-scale (1:250,000 to 1:1,000,000), and 7% continental-scale (>1:1,000,000). In some places, older regional-scale maps^3^ remain the best legacy information available. Wherever possible the geological classification used the best, highest resolution maps available. The final classification of geological unit polygons cites 234 source maps. Of the original 68,381 ADD polygons, only 7920 remain in the derived GeoMAP dataset with the same position and geometry. The positional accuracy of final polygon boundaries is strongly dependent on the quality and scale of the original legacy maps, but for the most part it is around ±250 m.

Although no new fieldwork was completed specifically for GeoMAP purposes, access to sub-metre scale satellite imagery and trimetrogon aerial (TMA) photographs was obtained through Google Earth (https://www.google.com/earth/) or the U.S. Polar Geospatial Center (PGC) (https://www.pgc.umn.edu/data/aerial/). These enabled some new observations and interpretations to be made at high-resolution, or for reasonable inferences to be transferred from one nearby outcrop to another. Regardless, a surprising number of places in Antarctica have had no geological observations and lack high-resolution imagery, resulting in local gaps and uncertainties in geological mapping. The question symbol ‘?’ was used for 4207 polygons in the dataset to indicate places where the exact nature of rock outcrops or their age is still unknown and future work is recommended.

## Data Records

GeoMAP data are available for download directly from PANGAEA^24^, or can be accessed through webmaps and webservices delivered by GNS Science^33^. GeoMAP uses GIS methods to store, manipulate, and present bibliographic and geological information. There are four main feature classes (Table 1) in the archived ArcGIS geodatabase and QGIS GeoPackage. The database also contains a geological legend (feature dataset) that portrays a time-space diagram. A general overview of these data is provided here, but more-complete information, including tables of all attributes and attribute field content, is provided in metadata records^33^ and some online documentation (https://geomap.readthedocs.io/en/latest)_._

**Table 1:** Summary of the main GIS feature classes in the GeoMAP v.2022-08 dataset.

ATA_GeoMAP_Feature Class	No. & Type	Description	Attribute Fields
sources	589 polygons	Spatial bibliography of key legacy geological maps that were consulted during GeoMAP construction. Provides link to sources used in geological_unit classification.	17 different fields describing the source’s author(s), title, publication, year, institution, scale of map, type of publication, and lead national Antarctic program.
geological_units	99,080 polygons GeoSciML-Lite	Geological unit data for Antarctica. A synthesis of existing regional mapping of geology in a single dataset at continental scale. Polygons cover exposures of both bedrock and unconsolidated cover deposits (such as glacial tills), but also include areas of ice from re-frozen meltwater.	42 fields describing the unit type, name, age, lithology, stratigraphy, and supporting background information, where known. Each is linked to legacy maps through a sources attribute. Can be used to generate a variety of categorical classifications.
faults	1,784 lines GeoSciML-Lite	Fault data for Antarctica. A synthesis of existing published and unpublished mapping in a single dataset at a regional scale.	32 fields describing the locational accuracy, exposure, activity, type of fault, and sense of movement and displacement, where known. Also fields describing scale of data capture and bibliographic source.
quality	43,201 polygons	Areas covering 1° longitude and 15 minute latitude intervals, categorized by a subjective rank of quality for GeoMAP geological unit and fault data within the area. Also provides binary indication of whether there are geological units within the polygon.	5 fields including a numerical rank of quality from 1 (low) to 5 (high), with other contextural comments such as progress of data capture, work outstanding.
legend_geological_units	451 polygons Chronologic grids and annotation are also provided with the feature dataset	A geological legend portrayed as a time-space diagram illustrating the representative age and types of rock at the approximate longitude where exposures of that geology occur. Constructed using EPSG:4978 (WGS 84) these data are also suitable for projection in EPSG:3031 (WGS 84/Antarctic Polar Stereographic).	11 fields to match the ATA_GeoMAP_geological_unit feature class. Includes mapsymbol, sourcecode group name, simplecode lithology, stratigraphy, and tectonic province.

### Source map bibliography

The ATA_GeoMAP_sources_poly feature class is a spatial bibliography of polygons representing the geological maps consulted or directly used in construction of GeoMAP. Each polygon defines the spatial cover of maps and has attributes describing the source’s authors, title, publication, year, program and scale of publication. The data structure complies with the GeoSciML 4.1 standard and uses the relevant Common Gateway Interface (CGI) controlled vocabularies^26^. A unique identifier SOURCE is also used to link geological polygons in ATA_GeoMAP_geological_units to the bibliographic reference used for their definition.

### Geological mapping units

Delineating geological units is a fundamental part of the geological mapping process. Geological maps are typically based on lithostratigraphy, biostratigraphy, age, and rock types, or combinations of these. A combined chronostratigraphic and lithostratigraphic approach was adopted for GeoMAP, in which the description of areas is based on dominant age and/or rock type. To be depicted the geological unit must have sufficient thickness and/or scale, which is typically greater than 10–20 metres thick or 200 m across for GeoMAP. Any units thinner or narrower than this are likely to have been omitted, unless particular emphasis on the unit is warranted through a need to highlight features of some special scientific significance^22^.

Geological polygons have been stored in the feature class ATA_GeoMAP_geological_units, the principal output from the project. These vector data comply with the GeoSciML Lite standard for GeologicalUnitView, and fields required by that standard were populated using the CGI vocabulary (v2016.01)^26^. Polygons each have 42 populated fields holding attributes regarding stratigraphic nomenclature and hierarchy, age, lithology, and primary data source, etc. Perhaps the most important attribute fields are SOURCECODE and MAPSYMBOL.

SOURCECODE is the classification initially assigned by a legacy map author (source) pulled directly from the SOURCE publication, following whichever convention was used by the author(s) in their original publication. SOURCECODE values commonly follow conventional geological labels (1–2 characters indicating age followed by 1–2 characters indicating lithology) or they can be a number or character-number combination like a sample identifier. Question marks (?) have been placed in the symbol to indicate where there is uncertainty in the original author’s identification, or that an area has yet to be mapped beyond reconnaissance level, and geology inferred. SOURCECODE provides a bibliographic link that enables initial hard-copy maps to be retrieved or reproduced locally in a digital format. The 993 distinct values in SOURCECODE, however, are not a useful way to classify the entire continent.

**Fig. 2:**
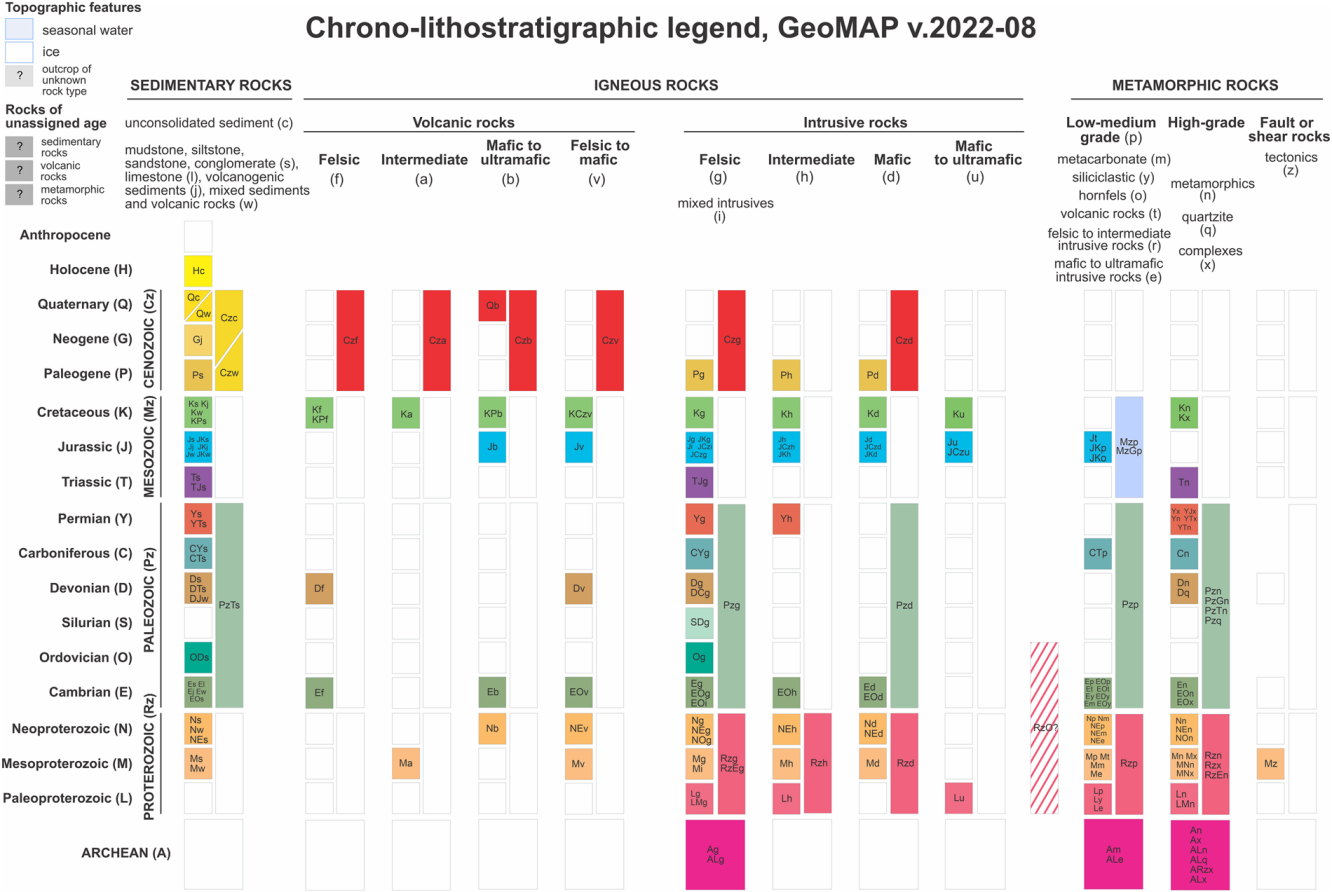
The chronostratigraphic and lithostratigraphic convention adopted within the GeoMAP ATA_geological_units layer by the attribute MAPSYMBOL to unify all the different geological studies across the continent. Coloured boxes on this chrono-lithostratigraphic legend show symbols present in the dataset, as used in Fig. 1, whereas clear boxes are not present (or have yet to be assigned). Note that geological units which span multiple time periods have symbols showing the oldest and youngest time periods. e.g. Cambrian to Ordovician sedimentary rocks = EOs; Paleoproterozoic to Mesoproterozoic high-grade metamorphic rocks = LMn.

By way of contrast, the MAPSYMBOL code provides GeoMAP’s main classification of the geological identity of the polygon, with values restricted by a formatting convention defined by the GeoMAP chrono-lithostratigraphic legend (Fig. 2). GeoMAP has adopted a convention that is very similar to the 1:1 million geological map of Australia^34^. CAPITAL letters are used to represent age (Chronostratigraphic subdivision, also used for the AGECODE field) and small letters representing lithology (rock-type as a lithostratigraphic classification, also provided in a LITHCODE field). The MAPSYMBOL unifies the original SOURCECODE and classifies polygons consistently across the entire GeoMAP dataset into 186 different units (Fig. 2). A cartographic layer file symbolised according to this chrono-lithostratigraphic subdivision is provided for a spatially projected legend layer in the GeoMAP data download package.

The attribute field NAME provides either a textual name of the rock unit, or simplified type of rock. Where possible the formally defined and published stratigraphic name is assigned to the polygon, for example, as per the lexicon in the geologic map of southern Victoria Land^22^. However, many units have been named informally or only classified by lithology. For the most part, NAME has generally been derived from the SOURCE publication and follows whichever convention was established in the legacy work by the original authors. There are 789 unique NAME values.

A POLYGTYPE attribute with restricted fields of ‘rock’, ‘moraine’, and ‘ice’ is the simplest possible classification of the geological unit polygons. There are occurrences of water, or ice that have re-frozen from meltwater, throughout Antarctica. Seasonal accumulations of supraglacial water, small ponds, and lakes are commonly re-frozen into clear dark ice that can be distinguished from blue ablation ice in remotely-sensed imagery and digital surface models by their darker/clear colour and/or a flat surface. Many are defined in the ADD lakes dataset^27^, but lots of new occurrences are added in GeoMAP, since quantification of surface meltwater is likely to be a feature of importance for understanding Antarctica’s future^5,15^. There are 3383 ‘seasonal water’ polygons in the dataset within the POLYGTYPE = ice. For GeoMAP they were manually mapped, using either the ADD_lakes_high_res_polygon.shp or satellite-based observations, predominantly from LIMA. Other projects are now working towards automated machine learning approaches^15,35,36^ to ensure that future changes and variations in seasonal water are tracked.

A more basic geological classification can also be achieved using the LITHCODE attribute field derived from the lowercase letter of MAPSYMBOL (Table 2). A GIS layer file ‘Simple Lithology’ provided in the GeoMAP data download package classifies the geological unit feature into 8 classes. Alternatively, there is an attribute field SIMPLECODE to provide a generalised chronological and lithological subdivision into 21 classes (Table 3). These are expected to provide a more-useful definition of the variation in age, rock strength, and chemistry without going to the full 186 unit chronostratigraphic and lithostratigraphic classification provided by MAPSYMBOL.

**Table 2:** Basic grouping of the geological unit polygons can be achieved using the lithostratigraphic LITHCODE attribute (lower case letter of the MAPSYMBOL). A cartographic layer file of ‘Simple Lithology’ is provided in the data download package.

LITHCODE	SIMPLE LITHOLOGY DESCRIPTION
	Seasonal ice and water
c	Unconsolidated sediment
j,l,s,w	Sedimentary rock
a,b,f,v	Volcanic igneous rock
d,g,I,u,h	Intrusive igneous rock
e,m,o,t,y,p	Low-medium grade metamorphic rock
n,q,x,z	High-grade metamorphic rock
?	Unknown or unclassified rock

**Table 3:** A simple classification of the geological unit polygons using the SIMPLECODE numerical attribute that distinguishes SIMPLECLASS and SIMPLEDECSRIPTION. A rendered layer file ‘Simple Geology’ is provided in the data download package.

SIMPLECODE	SIMPLECLASS	SIMPLEDESCRIPTION
10	OTHER	Seasonal water and ice
11		Unknown or unclassified rock
20	QUATERNARY-NEOGENE	Unconsolidated colluvium, talus, alluvium, and undifferentiated till
21		Youngest glacial gravel, till, and supraglacial material (Holocene)
22		Unconsolidated coastal ice shelf till, beach, or lake deposits
23		Older glacial gravel and till (Miocene-Quaternary)
30	CENOZOIC	Sedimentary rock with interbedded volcanic or volcaniclastic rock
31		Volcanic rock - basalt to rhyolite lava flows and pyroclastic material
32		Intrusive rock - granite, granodiorite, gabbro, or syenite (Eocene-Oligocene)
40	MESOZOIC-CENOZOIC	Sedimentary and volcanic rocks, pyroclastic material (Jurassic to Paleogene)
41		Volcanic rock (Jurassic-Paleogene)
42		Ferrar Igneous Province and related volcanic and sedimentary rock (Jurassic)
43		Silicic volcanic and continental sedimentary rock (Jurassic)
44		Unmetamorphosed granitoid, gabbro, and other intrusive rock
45		Metamorphosed gneiss and migmatite (Triassic, Cretaceous)
50	PALEOZOIC-MESOZOIC	Beacon Supergroup and other sandstone-rich sedimentary rock (Devonian-Triassic)
60	PROTEROZOIC-PALEOZOIC	Intrusive rock - granitoid, diorite, gabbro, and orthogneiss
61		Folded low-grade metasedimentary and metavolcanic rock
62		Low to medium-grade metamorphic rock - schist, marble, metavolcanic, metasandstone
63		High-grade metamorphic rock - orthogneiss, paragneiss, schist, and amphibolite
70	ARCHEAN	Metamorphic and intrusive rock - schist, gneiss, granulite, migmatite, and anatectite

### Faults

The feature class ATA_GeoMAP_faults contains a series of lines that mark places where faults or shear zones have been observed to cross-cut geological units, forming boundaries between adjacent polygons, or interpreted as concealed sub-ice discontinuities based on otherwise unexplained changes in geology. Antarctica appears relatively unique in that there seem to be very few outcrops with fault or fault-rock exposures compared with ice-free continents, though examples do occur^37,38^. Perhaps this is because fractured-rock is more easily eroded than unfractured-rock, the crushed rock in fault-zones tends to coincide with gulleys and topographic low-points, and these preferentially fill with, and become obscured by, wind-blown snow and ice. Most legacy maps depict some form of faults beneath snowfields and glaciers, many of which are interpreted from changes in geology or geophysical characteristics. There are few studies, however, with accompanying documentation that rationalises the basis for interpreting such structures, fault-type, sense of motion, or scale of displacement. Many may simply reflect a geological paradigm applied at the time or were used to rationalise lack of information for understanding local geological relationships and structure.

The compiled faults feature class contains 1784 lines (arcs) each with 32 attribute fields to describe the locational accuracy, exposure, activity, type of fault, orientation, and the sense of movement, where this is known. It complies with the GeoSciML Lite standard for ShearDisplacementStructureView with fields required by that standard populated using the CGI vocabulary (v2016.01)^26^. As it contains many interpreted structures, and some that may be speculative and debatable features, all lines within the ATA_GeoMAP_faults feature class are linked to the original bibliographic reference where they were first defined using the unique SOURCE identifier in the ATA_GeoMAP_sources_poly spatial bibliography. The faults feature class includes some uncertain ‘tectonic province boundaries’, concealed beneath the ice, that have been inferred at a continental-scale^23^.

### Chrono-spatial legend

**Fig. 3:**
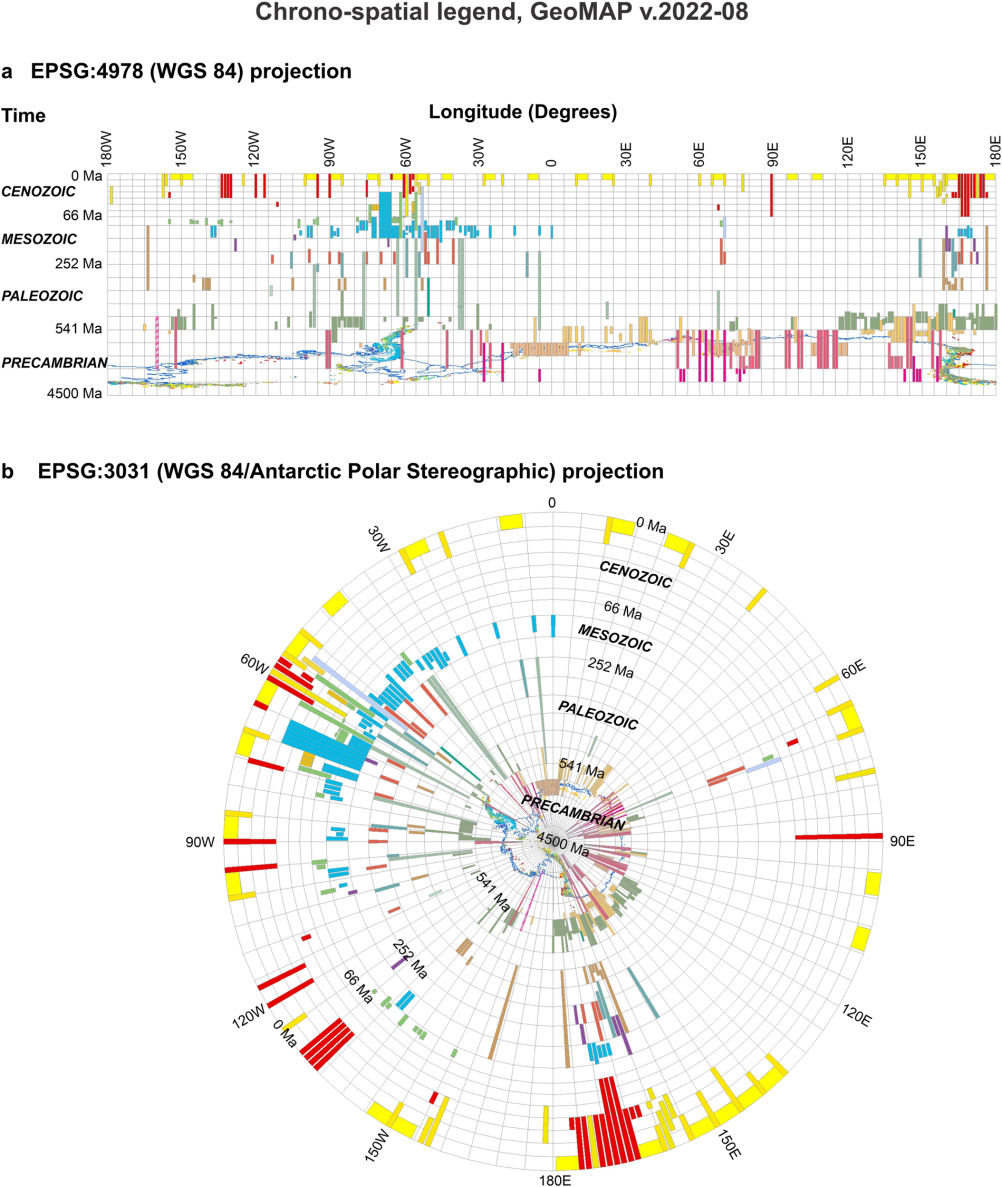
A unique chrono-spatial legend is provided as a feature dataset in GeoMAP v.2022-08 that can be displayed with different projections shown here with ‘thumbnails’. (**a**) The geological units on a time versus longitude graph using the EPSG:4978 (WGS 84) coordinate system. (**b**) The exact same data using EPSG:3031 (WGS 84/Antarctic Polar Stereographic) projection. Both follow the chrono-lithostratigraphic colour scheme used in Fig. 2 to colour the ATA_GeoMAP_geological_units dataset. This feature class has eleven geological fields that can be explored with the same attribute queries as used to select and find geological map unit polygons. Although it is impossible to show the detail of these diagrams in a journal-sized figure, they serve to indicate functionality provided by these data in a GIS program.

An innovative feature of GeoMAP v.2022-08 is a geological legend portrayed as a time-space diagram (Fig. 3). The GIS feature dataset ATA_GeoMAP_legend was created to show geological map units by age in the approximate longitude at which exposures occur around Antarctica. Each polygon in the chrono-spatial legend has attributes derived from ATA_GeoMAP_geological_units enabling it to be coloured by MAPSYMBOL or SIMPLECODE. Alternatively, they can be queried and selected by any of the eleven attribute fields. Legend polygons also record the code used on the original published source map or dataset using the SOURCECODE attribute.

As the name implies, the chrono-spatial legend is a time-space graph showing the spatial location of objects as a function of time. Similar to most conventional geological legends, it shows time running up the diagram equivalent to a Y-axis, so the bottom is the past, or older times, and the top is the present, or more recent times. Lines of longitude form an X-axis and provide the geographic context to anchor features in the legend data layer. The chrono-spatial legend was constructed using EPSG:4978 (WGS 84) coordinate system but is suitable for projection in other coordinate systems. Popular GIS software packages, such as ArcGIS and QGIS allow a user to project geospatial layers “on the fly”. Once the geological legend is loaded in a map project, by changing the coordinate system in the map properties, it is possible to see the spatial context of different rock ages from a different perspective. A user might switch between EPSG:4978 (WGS 84) or EPSG:3031 (WGS 84/Antarctic Polar Stereographic) or EPSG:3832 (WGS 84/PDC Mercator) coordinate systems to gain an appreciation of the geological age variation around the continent.

## Technical Validation

With positional accuracy of final polygons predominantly ±250 m, GeoMAP v.2022-08 reliably represents geology at 1:250,000 scale. However as well as the precision and accuracy of the polygons there are spatial variations in both the internal quality of interpretative information available for compilation of the dataset, as well as the degree of attention an area may have received by the GeoMAP team. One of the hardest tasks was, and still is, building consistency and capturing the local nuances of different interpretations available. There will undoubtedly be debate as to how well this has been achieved, but there has always been full expectation that GeoMAP should be a ‘live’ dataset that continues to evolve and improve over time.

**Fig. 4:**
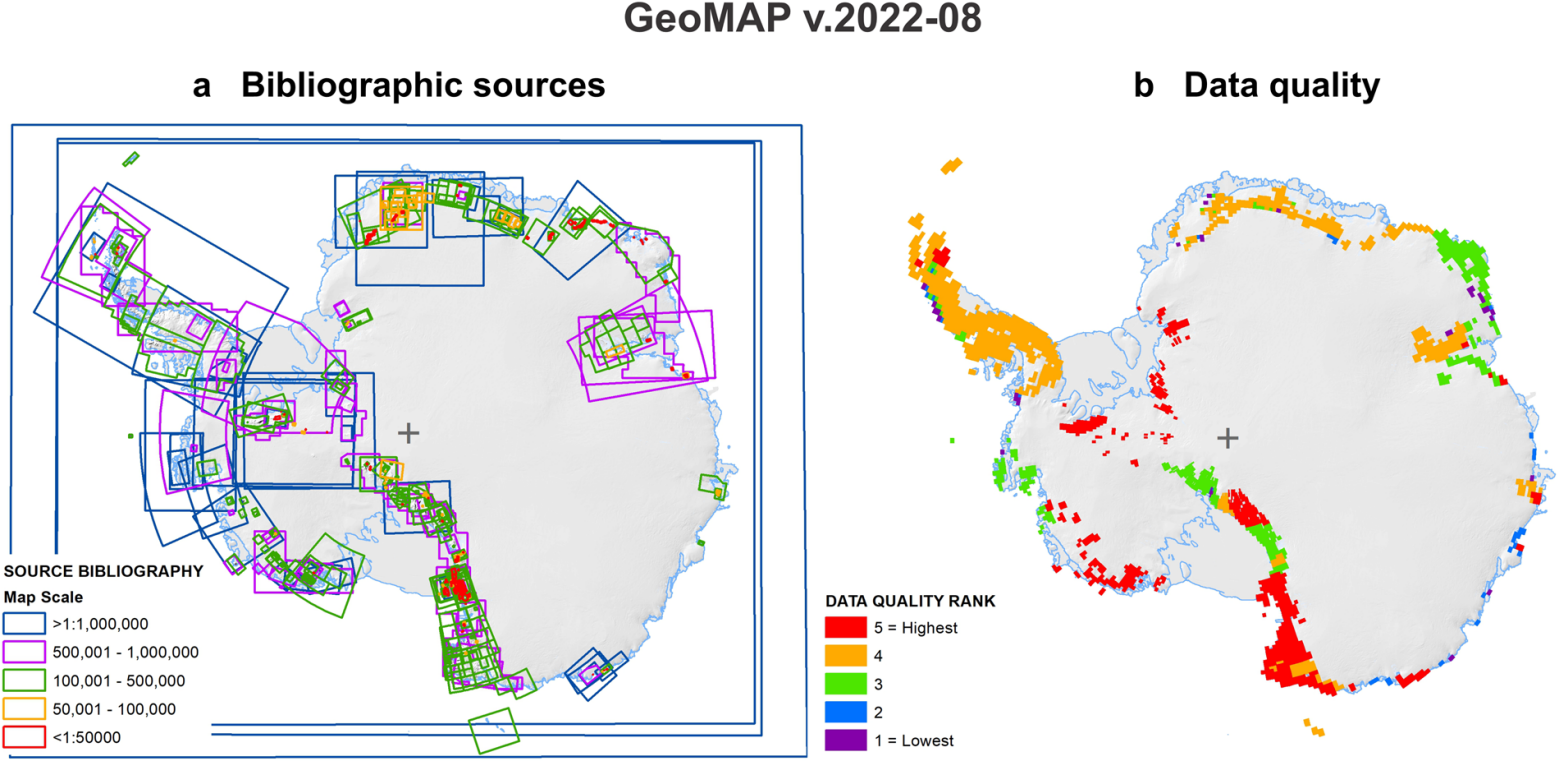
Bibliographic source and data quality for GeoMAP v.2022-08. (**a**) The distribution of bibliographic source maps used in the compilation of GeoMAP, with map area outlines coloured by scale. (**b**) The subjective ranking of the relative quality of the geological data, where different colours represent a subjective raking on a scale of 1 to 5 where there are rock outcrops. For a detailed description of the quality ranking see Table 4.

As GeoMAP was being constructed from continental- to regional- and to local-scales, it evolved in both the positional accuracy and level of detail of geology represented. The feature class ATA_GeoMAP_quality was generated across 1 degree longitude and 15 minute latitude intervals to provide a subjective rating of ‘quality’ and used to track GeoMAP progress. It subjectively rates availability of geological mapping, availability of other legacy information, the attention various areas have received by the GeoMAP team and regions where there is data yet to be captured (Fig. 4; Table 4). It is inevitable that some degree of geological complexity and variety of scientific approach and interpretations will have also inadvertently been included in the ranking, although this was not the primary intent of this feature class. As well as tracking progress for project management as the dataset evolved, the final published v.2022-08 quality feature class can be used to indicate confidence for future cross-discipline work and an impetus for GeoMAP improvements. A number of areas for improvement are also listed in the Usage Notes below.

**Table 4:** Outline of ATA_GeoMAP_quality ranking used to indicate variation in data veracity across a grid at 1 degree of longitude and 15 minute latitude, and to highlight regions that would benefit from further work or investigation.

QUALITY	DESCRIPTION
5 (Highest)	Bedrock geology has been captured from mapping at 1:250,000 or more detailed; cover sequences and occurrence of seasonal water have been reviewed and classified from ground studies, or aerial or satellite imagery; fault data have been captured; rock outcrop polygon areas have been checked against LIMA or other satellite imagery (accurate to ±100 m); links to a source bibliography are complete.
4	Bedrock geology captured from mapping at regional scale (mostly <1:250,000); cover sequences and occurrence of seasonal water have been reviewed and classified from aerial or satellite imagery, but there may not be local studies to constrain age; most faults have been captured; rock outcrop polygon areas have been checked against LIMA or other satellite imagery (accurate to ±200 m); links to a source bibliography are complete.
3	Bedrock geology has been captured from mapping at regional scale (mostly >1:250,000); review of cover sequences and seasonal water is quite limited and could be rationalised; some fault data have been captured; there has been some checking of rock outcrop polygon areas against satellite imagery (accurate to ±500 m); further bibliographic links to geological studies could potentially be added.
2	Bedrock geology may be well-constrained, or alternatively interpreted from large scale mapping (e.g. >1:1,000,000); there has been very limited review of cover sequences and seasonal water; fault data are unlikely to have been captured; rock outcrop polygon areas could be improved by checking against satellite imagery (only accurate to ±1000 m); links to bibliographic source information are missing, or the bibliography is incomplete.
1 (Lowest)	Quality rank refers to the early stages of work where little or no information had been attributed to rock outcrop polygons; and polygons have not been checked for their locational position and/or shape. In some places there is no information to assign (where outcrops may not have been visited or had geology inferred) in which case polygons have been attributed as ‘unknown’. There are still quite a few places in Antarctica where an entire 1° × 15 minute grid polygon appears to be unknown.
0	There are no outcrops of rock or surficial deposits in the grid polygon.

## Usage Notes

GeoMAP is licensed under a Creative Commons Attribution 4.0 International License and available for download through a data repository^24^. Acknowledgement of the source of data from both GNS Science and the SCAR GeoMAP Action Group is requested. It may be appropriate to cite both the data repository and this manuscript separately, depending on whether the data themselves are being used.

A goal of the GeoMAP Action Group has been to capture a digital dataset to first enable ‘cross-discipline science’, with a secondary intent to support ‘geological science’. But as the dataset evolved, there has been a natural tendency to add in and improve the geological information it contains, and shift focus towards the more-specialised geological end user. Although it was not originally conceived as a specialist reference dataset for geologists undertaking detailed research, it clearly provides context for local research. GeoMAP may well become a useful first port of call for introductory overviews and bibliographic links to original work, acting like a spatial ‘Wikipedia’ of Antarctic geology. During construction of GeoMAP v.2022-08 there were several areas specifically identified where the dataset can be improved in future versions, particularly for geology. Some are listed in detail below, while other suggestions for localised improvement are recorded in the COMMENTS attribute field in the ATA_GeoMAP_quality feature class.

### Cross-discipline application

The definition of rock and substrate composition has potential to inform ecological, environmental, biological, heat flow, and meltwater modelling, as well as many other cross-discipline studies. Of the ‘Priorities for Antarctic Research’ identified by the Antarctic Roadmap Challenges Project^39^ we have identified at least 22 of the 80 questions that can be informed by GeoMAP data, including: Ice sheet & sea level: Q27, 29, 32–34; Dynamic Earth: Q35–42; Life on the precipice: Q46, 48, 49, 54, 55, 68; Human presence: Q74, 75, 79). Future improvements in both the detail (scale) of mapping and bedrock, cover, and ice margin interpretation will help GeoMAP become more specifically useful for a wider range of scientists.

Classification can improve categorical reduction of chronostratigraphic and lithostratigraphic variability, and greatly simplify the use of geological data in cross-discipline interrogation. The most basic geological classification can be achieved using LITHCODE (Table 2) and the simple geology attribute fields (SIMPCODE, SIMPCLASS, SIMPDESC) group geological units into 21 age and rock type classes (Table 3). Alternatively, the ATA_GeoMAP_geological_units attribute TECTPROV provides a different break-down of polygons into more-interpretative tectonic provinces^23,40^. It is just one of many possible interpretations of cratonic blocks and tectonic belts^41–47^, which tend to be strongly reliant on available geochronology, geophysics, and correlation to neighboring continents. The functionality of GeoMAP and other digital geological data might well be improved if a high-level stratigraphic/structural classification scheme were able to be collectively agreed upon for Antarctic Geology, similar to that developed for New Zealand^48^.

### Geological context and high-level classification

There are many ways by which Antarctica can be subdivided into high-level geological units. Areas of outcropping geology are commonly classified into ‘crustal blocks’, ‘tectonic provinces’, ‘cratons’ or ‘orogenic belts’. These define parts of the continent based on age and orogenic history and group the rock’s unique modification by accretionary and collisional additions, or rift-driven subtractions. By combining field geology with geochronology, geochemistry, geophysics and knowledge of geological history of neighbouring continents, such subdivision can be projected beneath ice-covered areas. Major tectonic boundaries can also be constrained by geophysical interpretation and the relative location of different rock outcrops. However, classification of outcrop geology then becomes strongly dependent on this interpretative process and the availability and scale of geophysical data to do so. As a consequence, a plethora of continental-scale subdivisions^23,40–47^ have evolved as rapidly as the available science.

A deliberate feature of GeoMAP has been to focus on the capture of ‘known geology’ of rock and bare sediment exposures, rather than interpreted sub-ice features, in part because it is scientifically conservative and enables the observational dataset to become a longer-lived product. GeoMAP v.2022-08 does, however, provide the attribute field TECTPROV which contains one version^23,40^ of a high-level subdivision. There are clearly a number of other possible high-level subdivisions or more-detailed groupings. The Dronning Maud Land sector of East Antarctica, for example, has been subdivided into at least five other distinct units (Grunehogna Craton, Natal Belt, Maud Belt, Tonian Oceanic Arc Super Terrane and Lutzow Holm Complex)^32,41,44,46^. What could be really informative would be to capture different interpretations for a future GeoMAP, with the spatial resolution afforded in the GIS, using new attribute fields (e.g. TECTPROV.v1, TECTPROV.v2 etc.) in the ATA_GeoMAP_geological_units and ATA_GeoMAP_faults feature classes. Together with the latest geochronological and geophysical data, it may help scientific debate on the continent’s history and construction.

### Local geological detail

There are places where local detail might be easily improved with some basic geological mapping. Throughout northern and southern Victoria Land the flat-lying sequences of Beacon Supergroup have been subdivided from the Ferrar Dolerite sills that intrude them. Individual units within the Beacon Supergroup have also been distinguished in much of the dataset. However there remain some large areas in the central Transantarctic Mountains where the Beacon and Ferrar rocks are yet to be distinguished, despite being clearly visible in high-resolution satellite imagery or aerial photographs. It would be desirable to subdivide these in future iterations of GeoMAP as their distinction reflects important local variations in rock colour, albedo, substrate chemistry, rock competence and fracture density.

There has also been some local-scale geological mapping yet to be captured by GeoMAP v.2022-08. The Japanese National Institute of Polar Research, for example, have produced a series of 39 beautiful, detailed, hard-copy map sheets at between ≤1:25,000 to 1:250,000 scale from Japanese Antarctic Research Expeditions (JARE) to Dronning Maud Land. Some of their map units have been grouped and/or simplified for subsequent digital data^32^ and GeoMAP v.2022-08. Numerous small-scale geological maps at ≤1:50,000 scale have also been published locally within the South Shetland Islands^49–54^. However, these maps are discontinuous and recent works appear to contain some marked interpretational differences. The next version of GeoMAP may need a concerted effort and a collaborative approach to rationalise detailed geological observations into a simplified syntheses of these regions.

Perhaps most importantly, the design and data structure of GeoMAP easily enables local maps, or new mapping, to be captured to future versions and linked back to the original work through the bibliographic ATA_GeoMAP_sources feature class (Fig. 4a). It is hoped sufficient time can be invested for the spatial resolution of GeoMAP to be improved in future versions. There are also a number of areas where we have yet to find any legacy data or observations of geology at all, which are distinguished in the geological_units layer with a ‘?’ symbol and ‘unknown’ attribute. These are clearly places to target searches through unpublished archives, acquisition of satellite data, and/or future expeditions.

### Glacial processes and deposits

With strong polarity between onshore and offshore geological records of the Neogene in Antarctica, there is potential to greatly improve our understanding of the source of glacial deposits, cover sequences and past behaviour of ice. Although continuous records of climate exist offshore and have been a focus for two or three decades, they are expensive to collect and are spatially limited (total Antarctic drilling to date ~5 m^2^ in plan view). By way of contrast, onshore records are discontinuous but commonly visible and accessible, with 10 orders of magnitude greater spatial extent (outcrops = 5 × 10^10^ m^2^) than drilling. A logical next step for mapping of these glacial deposits will be to use GeoMAP to help classify high-resolution imagery and multispectral datasets^17–19^, then generate compositional maps at metre-scale resolution from which source areas can be inferred.

A specific focus of GeoMAP was to capture information on glacial sequences, which appear to be under-utilised in contemporary science related to climate change. To date, the mapping of glacial deposits and landforms has been mostly localised, and continent-wide geospatial data for glacial-geologic and meltwater features are lacking. Regional-scale maps have limited depiction of post-Miocene surficial geology and geomorphology, and have highly variable spatial reliability, but the joint use of maps and satellite imagery can provide a wealth of information for data mining. The second edition geological map of southern Victoria Land^22^ that was subsumed in GeoMAP, for example, recognises c. 70 units pinpointing the locations of deposits and indicating their mode of formation, age, and likely source, compared with two units (till and scree) mapped in the first edition^1^.

**Fig. 5:**
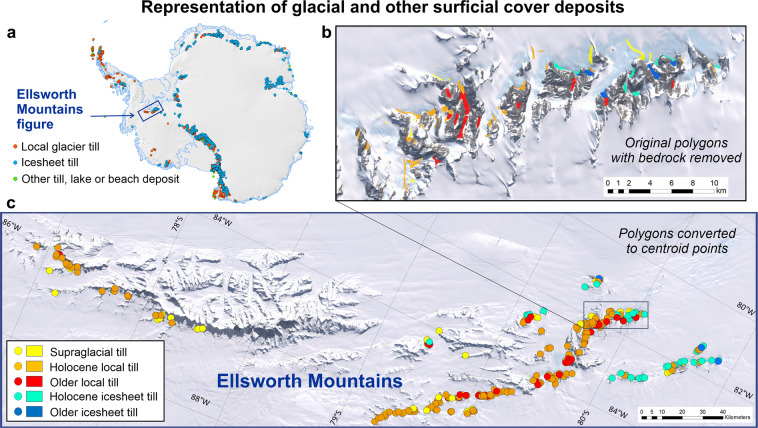
Extract of GeoMAP data from the 350 km-long Ellsworth Mountains. (**a**) Location diagram and overview of the distribution of cover deposits as centroid points. (**b**) An enlarged frame for part of the Heritage Range to exemplify the degree of detail of glacial deposit polygons recorded as tills in the ATA_geological_units layer. The polygons are depicted over the LIMA satellite imagery^29^, with bedrock geological polygons removed. (**c**) A wider regional-scale map in which geological unit polygons have been converted to centroid points, then displayed on the basis of geological NAME using the same colours as polygons in (**b**). It exemplifies one way in which data can be rendered to provide new perspectives of climatic processes on the glaciers.

To exemplify an application of regional data, maps of the Ellsworth Mountains have been generated with coloured polygons and dots depicting glacial till localities (Fig. 5). The higher-resolution inset map (Fig. 5b) has red and orange polygons showing location of tills from local glaciers, whereas blue and green dots represent tills left behind by the waxing and waning of ice-sheets during larger climatic events. Polygon centroids were used to generate simplified dot points (Fig. 5c). The Ellsworth Mountains have an impressive gradient in the distribution of these glacial deposits and landforms, with a complete absence in the higher central part of the range – where there is greater snowfall and katabatic winds are less extreme. In the search for ecological domains or places for life at the extremes, or hot-spots that will be first affected by climate change, GeoMAP has potential to elucidate regional-scale gradients and might be a useful first port of call.

Detailed investigations in southern Victoria Land^8^ enabled the regional geological maps to evolve from having just two cover sequence units^1^ to >70 units related to either the East Antarctic Ice Sheet, West Antarctic Ice Sheet, or a local alpine glacier source. Where possible, GeoMAP v.2022-08 has also classified or interpreted glacial deposits elsewhere, depending on whether they pertain to either local glaciers, or more major trunk glaciers and ice sheets. The intent was to distinguish features that might potentially relate to local fluctuations in precipitation from those related to major climatic events. Similarly, some interpretations have been made of the relative age of cover sequence units, based on elevation and position relative to the waxing and waning of nearby glaciers and ice. This classification work should be considered preliminary. It is hoped it will enable hypotheses to be developed and tested, or underpin work on the source of deposits^18,19^ and exposure dating. The age and source of glacial deposits is an area where focussed work can be expected to change and improve future versions of the GeoMAP dataset.

### Structural measurements

There are an infinite number of directions for true north from the South Pole, or 360 if measured in one-degree increments. Elsewhere in Antarctica, the direction of true north at a site is dependent on the longitude at that site. This can present a problem for the collection of dip and strike observations, or dip and dip-direction, that are the foundation of structural geology. Over 7500 structural observations of features such as bedding, foliation, faults, joints etc were collected from legacy data for GeoMAP. These features form discontinuities in the rock and tend to have an influence on the geomorphology of peaks, ridges and valleys forming the landscape or present in sub-ice topography. It was initially hoped to be able to display the azimuth, or dip direction relative to true north. However, we subsequently discovered there are major difficulties in rotating data relative to true north at a continental scale using the EPSG:3031 (WGS 84/Antarctic Polar Stereographic) projection. Different rotations applied in capturing the structural data in ArcGIS also meant that many azimuths have been found to be locally incorrect. These structural data need dedicated checking and editing before they will be of sufficient quality to be supplied routinely with GeoMAP, but are available (with caveats) on request to the corresponding author.

## Data Availability

GeoMAP v.2022-08 has been generated for ArcGIS (10.8.1) and QGIS (3.4) as geodatabase and geopackage material, using a GCS WGS 1984 geographic coordinate reference and WGS 1984 Antarctic Polar Stereographic projection. Data were developed manually, then stored in a GIS database developed, web-delivered and maintained by GNS Science in New Zealand. Software ArcGIS® has been used to create the GIS database^24^, but data can be exported in a variety of formats and compatible with most other GIS software. ArcGIS data are available from the PANGAEA data archive^24^, an ArcGIS REST service (https://gis.gns.cri.nz/server/rest/services/SCAR_GeoMAP/ATA_SCAR_GeoMAP_Geology/MapServer), or viewed through a webmap (https://data.gns.cri.nz/ata_geomap/index.html). A series of QGIS and Google Earth KMZ files, exported from the ArcGIS geodatabase layers, are also available from the archive^24^. The original data have been segmented into ten regions to keep KMZ files at a reasonable (<25 Mb) and useable size. GeoMAP documentation (https://geomap.readthedocs.io/en/latest/) has been generated using code deposited on GitHub (https://github.com/selkind/GeoMap).
